# Supplementary figures and images for: Nanoparticle coatings for controlled release of quercetin from an angioplasty balloon
Source: PLoS One. 2022 Aug 24;17(8):e0268307. doi: 10.1371/journal.pone.0268307 (PMC9401142; doi:10.1371/journal.pone.0268307)

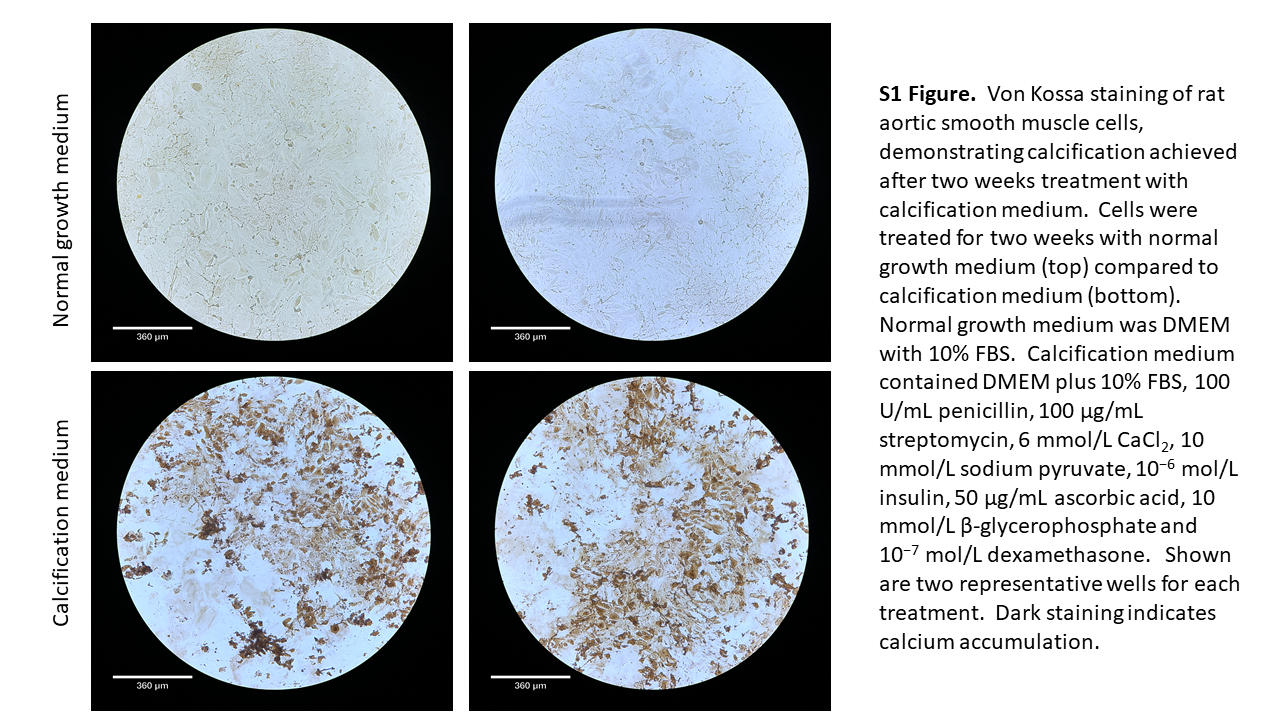

Supplement: S1 Fig — (TIF) [file pone.0268307.s001.tif]
